# Pediatric stroke and transcranial direct current stimulation: methods for rational individualized dose optimization

**DOI:** 10.3389/fnhum.2014.00739

**Published:** 2014-09-19

**Authors:** Bernadette T. Gillick, Adam Kirton, Jason B. Carmel, Preet Minhas, Marom Bikson

**Affiliations:** ^1^Department of Physical Medicine and Rehabilitation, Program in Physical Therapy, University of Minnesota, Medical SchoolMinneapolis, MN, USA; ^2^Alberta Children’s Hospital Research Institute, University of CalgaryCalgary, AB, Canada; ^3^Weill-Cornell Medical College, Burke Medical Research InstituteWhite Plains, NY, USA; ^4^Department of Biomedical Engineering, The City College of New York of CUNYNew York, NY, USA

**Keywords:** pediatrics, stroke, hemiparesis, modeling, transcranial direct current stimulation

## Abstract

**Background**: Transcranial direct current stimulation (tDCS) has been investigated mainly in adults and doses may not be appropriate in pediatric applications. In perinatal stroke where potential applications are promising, rational adaptation of dosage for children remains under investigation.

**Objective**: Construct child-specific tDCS dosing parameters through case study within a perinatal stroke tDCS safety and feasibility trial.

**Methods**: 10-year-old subject with a diagnosis of presumed perinatal ischemic stroke and hemiparesis was identified. T1 magnetic resonance imaging (MRI) scans used to derive computerized model for current flow and electrode positions. Workflow using modeling results and consideration of dosage in previous clinical trials was incorporated. Prior *ad hoc* adult montages vs. *de novo* optimized montages provided distinct risk benefit analysis. Approximating adult dose required consideration of changes in both peak brain current flow and distribution which further tradeoff between maximizing efficacy and adding safety factors. Electrode size, position, current intensity, compliance voltage, and duration were controlled independently in this process.

**Results**: Brain electric fields modeled and compared to values previously predicted models (Datta et al., 2011; Minhas et al., 2012). Approximating conservative brain current flow patterns and intensities used in previous adult trials for comparable indications, the optimal current intensity established was 0.7 mA for 10 min with a tDCS C3/C4 montage. Specifically 0.7 mA produced comparable peak brain current intensity of an average adult receiving 1.0 mA. Electrode size of 5 × 7 cm^2^ with 1.0 mA and low-voltage tDCS was employed to maximize tolerability. Safety and feasibility confirmed with subject tolerating the session well and no serious adverse events.

**Conclusion**: Rational approaches to dose customization, with steps informed by computational modeling, may improve guidance for pediatric stroke tDCS trials.

## Introduction

Transcranial direct current stimulation (tDCS) modulates human cortical excitability and improved motor outcomes in adults with and without neurologic diagnoses (Brunoni et al., 2012). For reasons of safety, cost, portability and potential promise for improved outcomes in children, we desired to investigate the application of tDCS in pediatric stroke. Considering the potential variability in dosing for the child’s brain, due to difference in brain size and anatomy, the direct transition from adult dosing to the safe and effective dose in a child has not yet been established. The relationship between the dose of stimulation (defined as the externally controlled parameters) (Peterchev et al., 2012) and brain current flow can be complex, such that computational models are used in dose design (Bikson et al., 2012). While for adults there are generally adopted principles regarding directions of effect (anodal excites, cathodal inhibits) and dosing (10–20 min, 0.5–2.0 mA), emerging evidence suggests even minor dose or brain integrity changes can lead to opposite effects (Fritsch et al., 2010; Fricke et al., 2011; Sohn et al., 2012; Batsikadze et al., 2013; Hasan et al., 2013; Schabrun et al., 2013). Moreover, modeling and imaging studies suggest limitations in the conventional effects of anodal and cathodal stimulation (Datta et al., 2009; Antal et al., 2012, 2014; Peña Gómez et al., 2012; Wagner et al., 2012; Rahman et al., 2013).

In contrast to individual-specific cortical excitability testing using TMS, subject-specific titration of dose in tDCS is rare, though probably equally important. When working with children, (even when adopting a technique such as tDCS, with a compelling safety record in adults) questions of safety gain new importance. Subject specific factors such as differences in brain size, water content, myelination, proximity of the brain to the skull and other characteristics of the developing brain may alter safety/tolerability and optimal dose (Minhas et al., 2012; Kessler et al., 2013). A broad review of available clinical reports integrated with computational models provides a basis to address this issue. This report describes the methods used in consideration of a pilot safety study applying a single-session of tDCS in a child with hemiparesis due to perinatal stroke and more broadly presents possible methodology for dose customization in pediatric populations (clinicaltrials.gov NCT01636661). A child who met the inclusion criteria for the pilot was identified, and the following methods were employed to establish safety and feasibility of the application of tDCS before the trial began. The description below identifies the methods in detail, while the trial itself is reported elsewhere (Gillick et al., in press). The study was approved by the University of Minnesota Institutional Review Board, and the Clinical and Translational Science Institute, Written and verbal parental consent and child assent was obtained. Modeling analysis of de-identified data at City College of New York of CUNY is IRB exempt.

## Components of trial design

**Figure 1:**
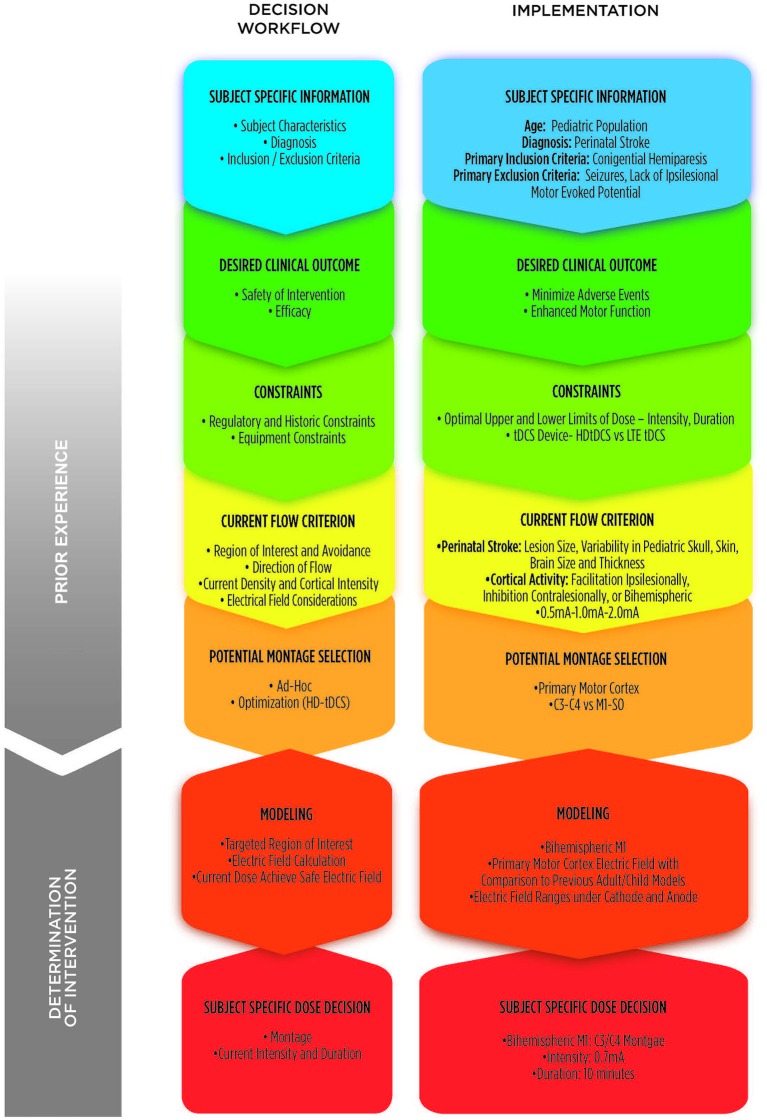
**Conceptual methodology for determination of dose**. Theoretical 7-step compartmentalization of decision workflow (left column) and implementation considerations specific to this case study of a 10 year-old child with hemiparesis due to perinatal stroke (right column). Incorporation of prior experience in the dose decision process coupled with modeling allows value-determination of subject-specific dose decisions for implementation.

At this time, a standardized tDCS dose has not been established, neither in adults or children. Understanding this, in order to determine tDCS parameters for this subject, we reviewed existing clinical experience and publications regarding current parameters of tDCS dose (including current polarity and intensity) from healthy adults and adults with stroke. Computational models were used to relate brain current flow in these cases to that in a child with stroke and resultant hemiparesis. Broadly, the goal was to use a stimulation paradigm that (1) produced the same brain current intensity (not necessarily same external dose) as in the adult cortex; and (2) targeted the motor cortices of the brain and the interactions between the hemispheres. However, a detailed methodological analysis, driven by rational trial design, and divergent approaches toward this aim must be selected balancing tradeoffs between innovation/conservatism, efficacy/safety, and putative non-monotonic dose response. Our process involved seven specific steps from concept to implementation: (1) gathering of subject-specific information; (2) formulating the desired clinical outcome; (3) considering constraints that may influence decisions; (4) defining brain current flow criterion; (5) investigating potential montages; (6) modeling montages to estimate brain current flow; and (7) determination of subject-specific dose. These stages are elaborated below and summarized in Figure 1.

## Subject-specific information

This pilot study focused on a 10-year-old child with a diagnosis of arterial perinatal ischemic stroke. The child had a normal perinatal history but presented in infancy with hemiparesis and was found to have a focal infarction (Kirton, 2013). Magnetic resonance imaging (MRI) confirmed a distal M1 segment of the middle cerebral artery stroke with involvement of peri-Rolandic regions of the right frontal and parietal lobes and centrum semiovale but sparing of the basal ganglia. Left hemiparesis was first noted at 4 months of age. At 10 years, the child had moderate hemiparetic cerebral palsy with a Manual Ability Classification System Scale Score (Eliasson et al., 2006, 2007) of II—“Handles Most Objects but with Somewhat Reduced Quality and/or Speed of Achievement”. The lower extremity was less affected, spasticity was minimal, and she had not received any new rehabilitation treatments within 6 months. She was otherwise developmentally normal, did not have epilepsy, and was not taking any neuroactive medications. Informed consent was obtained as part of a pilot safety and feasibility study on the application of tDCS in children with congenital hemiparesis (ClinicalTrials.gov Identifier: NCT01636661).

## Desired clinical outcome

Emerging evidence combining animal and human studies has defined models of developmental motor plasticity following perinatal stroke. These models suggest that inhibition of the non-lesioned hemisphere might enhance motor learning in the lesioned hemisphere, possibly via effects on excessive ipsilateral projections or disordered interhemispheric inhibition. Therefore, while the underlying neurophysiology is likely different, the strategy in adult stroke of inhibiting the non-lesioned hemisphere with non-invasive stimulation to enhance therapy may be applicable to children with perinatal stroke-induced hemiparesis (Hsu et al., 2012; Marquez et al., 2013). Studies in non-invasive brain stimulation and specifically in the use of repetitive transcranial magnetic stimulation (rTMS) have recently shown promising results in children with stroke, requiring further research to determine further clinical merit in this population (Kirton et al., 2008, 2010; Gillick et al., 2013).

## Constraints

### Dose

Given the volume of safety/efficacy data using a limited set of standard montages (Nitsche and Paulus, 2000; Brunoni et al., 2012), and because incremental changes may allow precise detection of potential adverse events, a historical approach of previous research and modeling was deemed appropriate. To ensure safety and to decrease the likelihood of side effects, adult protocols have typically utilized 0.2–2.0 mA with duration of stimulus ≤30 min (Rothwell, 2012; Batsikadze et al., 2013). However, we propose that when initiating studies in novel, potentially vulnerable populations (such as children) and/or injured brain (such as following stroke) approaches in adults should be re-examined using the latest available tools and scientific data to adjust for developmental variation in children. Furthermore, identification of possible risks and risk mitigation were identified (Table 1).

**Table 1:** **Possible adverse events related to transcranial direct current stimulation (tDCS) and risk mitigation**.

**Study procedure**	**Anticipated risks**	**Risk mitigation**
tDCS	Burn- Electrolysis	Ensure proper electrode contact with skin
tDCS	Stimulation in subjects with reduced sensation	Assess sensation, avoid placing electrodes over areas of decreased sensation
tDCS	Stimulation over broken skin, reduced resistance	Assess skin integrity, avoid placement of electrodes over recent shaving, skin defects
tDCS	Stimulation over conductive implants	Screen appropriately for exclusion criteria of implants
tDCS	Stimulation over a tumor which may alter metabolic activity	Screen appropriately for exclusion criteria of neoplasm.
tDCS	Threshold altering pharmacologic agent	Physician review of each medical record for determination of appropriateness for study inclusion.
tDCS	Itching, Tingling, Burning Sensation in the area of the electrodes	Ensure proper contact of surface electrodes with skin. Maintain current dosage within low-range of researched dosages. Ensure that electrode sponges are properly sanitized and that saline solution is appropriately employed.
tDCS	Headache	Ensure that headband securing electrodes is in proper placement, yet not to the level of impingement of scalp area. Maintain current dosage within low range of delivery.
tDCS	Pain- Neck, Scalp	Ensure that electrodes are in proper contact with skin and adjust head position as needed for comfort.
tDCS	Skin Redness	Ensure proper electrode position and proper level of moisture to even stimulation across the electrode
tDCS	Fatigue, Sleepiness	Screen for continuous effect at follow-up visit.
tDCS	Concentration or Mood changes	Evaluate cognitive status through physician examination and psychometric testing at three time points.

### Device

We used a Soterix Limited Total Energy (LTE) 1×1 tDCS unit which is limited to maximal current of 1.5 mA and 20 min maximum duration of stimulation. For this specific modeling, we first determined our current needs, and then the device model. The LTE also has a built-in sham stimulation mode, providing ramp-up and ramp-down stimulation at the beginning and end of the placebo session. During the 30–60 s period of time, the current is increased in order to reach the targeted dose and then discontinued. Two additional features reinforce safety. (1) An adaptive impedance monitoring feature provides continuous visual indication of electrode quality before and during stimulation. If impedance increases, producing a typical high voltage, the device not only automatically reduces the current output in accordance with changes in resistance but also provides an alert; and (2) a current monitoring feature acts as an independent current meter and provides continuous visual indication of the instant output of the device. This component adds redundancy to the safety of device and confirms the current setting. Pediatric size EasyStraps were used to ensure proper contact with the skin for each individual. Tolerability was enhanced by the RELAX feature which allows, if enhanced discomfort is expressed, transient reduction in current without interrupting or aborting the trial. Once comfort is re-established, the current may then again be adjusted. The device is limited to two electrodes and used with 5 × 7 EasyPads providing further dose design constraints (i.e., number of electrodes, electrode size and maximum current).

## Current flow criterion

Transcranial direct current stimulation alters brain function by polarizing the brain (Nitsche et al., 2008; Rothwell, 2012). Generally, brain regions exposed to higher current densities would be more likely to be influenced and each brain region receives various current intensity depending on the electrode montage. It is precisely because brain current flow is not a simple function of electrode montage (e.g., current is not restricted to only under the electrodes) that current flow criterion is described in terms of brain targets for neuromodulation, rather than scalp electrode position. Moreover, the intensity of current reaching the brain for any given applied electrode current can vary significantly depending on the montage and subject anatomy. Thus while electrode dose is controlled at the head surface (current in mA provided and montage), current flow criterion indicates the desired current intensity at the brain target level (in terms of electric field in units of V/m or A/m^2^).

With limited research in children, we recognized the need to re-examine adult-based practices including the effects of relatively high dose stimulation in adults. In deciding on desired brain electric field intensity it is typical to reference “gold-standard” experimentation where modulation of Transcranial Magnetic Stimulation (TMS)-motor evoked potentials (MEPs) by tDCS was quantified—with the strong caveat that TMS-MEP modulation and behavioral changes are only putatively linked. The M1-S0 montage conventionally used in these studies is shown to produce lasting TMS-MEP changes following several minutes of stimulation and with polarity-specific changes at intensities at 1 mA (Nitsche and Paulus, 2000)—where 1 mA corresponds to approximately 0.3 V/m of electric field in motor regions in adults (Datta et al., 2012).

Importantly, recent findings suggest that increasing tDCS current intensity may change the direction of these effects (Batsikadze et al., 2013). For example, in a study of 21 healthy adults, 2.0 mA cathodal (0.6 V/m brain electric field) tDCS for a duration of 20 min over the motor cortex resulted in enhancement of cortical excitability, *not* inhibition. An ongoing investigation of the application of tDCS in children and adolescents ages 10–18 reports that 1 mA anodal and cathodal stimulation over the motor cortex *both* produced an increase in the amplitude of the MEPs (Moliadze et al., 2013) which may reflect the higher brain current densities (e.g., ~0.6 V/m) produced in children for the same total current (see Section Modeling below).

Though non-linear (non-monotonic) TMS-MEP dose response is observed at higher tDCS intensities, at least across “moderate” tDCS stimulation intensities response seems consistent, at least for healthy inactive motor cortex. A study of 14 healthy adults investigated anodal tDCS over the motor cortex for 10 min at three different intensities—0.8, 1.0 and 1.2 mA—and found no difference in modulation of cortical excitability or inhibition (Kidgell et al., 2013a). One can therefore speculate that approximately 0.3 V/m for several minutes is a reasonable approach. Changes are polarity dependent, with anode/cathode tending to produce increased/decreased TMS-MEP amplitude, respectively. Importantly, increasing intensity or duration does not necessary magnify effects and the direction of changes can reverse (e.g., 2 mA cathodal is excitatory). Our decisions were (1) to limit total current to 2 mA or less based on skin tolerability and to use electrodes validated for tDCS; and (2) to limit current further as required to match electric fields corresponding to approximately 1 mA in adult (current flow criterion).

## Potential montage selection

Potential Montage Selection involves a selection of candidate montage to explore further with computational modeling. This involves integration of prior clinical trials and modeling; (1) with the subject specific information, desired clinical outcome, constraints and current flow criterion (Figure 1).

For our study two candidate montages were explored leveraging computational models to target the primary motor cortex (M1): (1) a supraorbital montage (M1/SO); and (2) a bihemispheric montage using the International 10/20 EEG System designation (C3/C4). Considering the M1/SO montage, the cathode would have been placed over the contralesional motor cortex and anode over the ipsilesional supraorbital region with the intent to inhibit contralesional effects upon the ipsilesional cortex. The bihemispheric montage was considered with the cathode placed over the contralesional cortex to down-regulate excitability, and the anode positioned to facilitate excitation of the ipsilesional cortex. An individualized head model was developed based on the child’s 1 mm^3^ resolution T1-weighted MRI scans obtained from a Siemens Trio Scanner with a 12-element head coil using methods described previously (Datta et al., 2012, 2013; Marquez et al., 2013).

## Results

### Modeling

**Figure 2:**
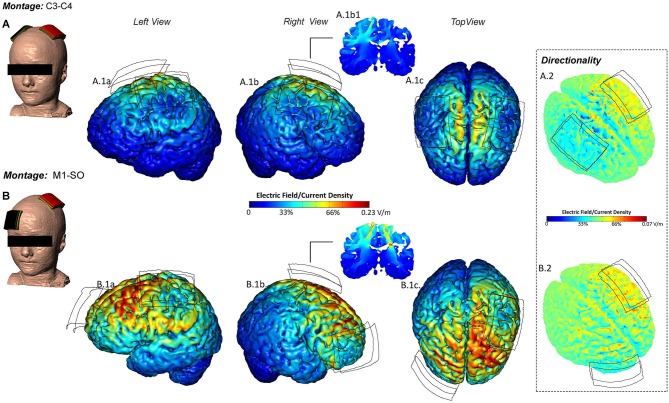
**Current flow predictions during tDCS in individual pediatric model for the M1-SO and Lateralized Temporal montages**. *M1-SO*- The center of anode (red) was positioned on the motor strip and the cathode (black) was positioned over the contraletral supraorbital area. At 0.7 mA applied current, the peak electric field was 0.23 V/m. *C3-C4*- The center of anode (red) was positioned over the left temporal lobe and the cathode (black) was positioned contralateral to the anode (M-O). At 0.7 mA applied current, the peak electric field was 0.29 V/m. EF plots in the left, right and top views, are shown respectively **(A.1a–c, B.1a–c)**. Cross-sectional coronal electric field plots were taken from the center of the anode **(A.1.b1, B.1.b1)**. Directionality plots were also plotted. The red corresponds to current flowing inwards, the green corresponds to a net flow of zero, and the blue corresponds to current flowing outwards **(B.1–B.2)**.

Consistent with previous models in adult and children, use of two large electrodes produced diffuse current flow between and under the electrodes (Bikson et al., 2012; Figure 2). The current flow pattern produced in our subject was, in this sense, broadly consistent with the typical current flow patterns produced by the M1/SO and C3/C4 montages used in prior clinical trials. The peak electric field produced in the regions-of-interest (under the electrodes) as well as across the entire brain (as using two pads typically produces peak electric field between electrodes) was compared for this subject and adapted from prior adult and pediatric models (Table 2). As noted previously, there are large (several fold) differences in peak electric fields even among adults (Datta et al., 2012; Edwards et al., 2013). The peak regional and global electric fields in our subject were comparable to those in previously modeled children, which are moderately higher than those in previously modeled adults (though comparable to the most sensitive adults). As modeled, application of 0.7 mA in our subject would produce peak electric field comparable to an average adult receiving 1.0 mA with a comparable distribution of current flow.

**Table 2:** **Electrical field (EF) ranges and peaks, in volts per meter, for each modeled head, by montage**.

		Montage		
		M1[A]–SO[C]	Lateralized motor C3[A]–C4[C]	Modeled sponge size
Child 1 (Normal Anatomy)	EF Range (C)	0.11–0.27	0.25–0.37	5×5 sponge pads
	EF Range (A)	0.14–0.30	0.26–0.44
	EF Peak	0.33	0.44
Child 2 (Normal Anatomy)	EF Range (C)	0.08–0.31	0.16–0.40	5×5 sponge pads
	EF Range (A)	0.18–0.44	0.19–0.40
	EF Peak	0.44	0.40
Child 3 Clinical Hemiparesis	EF Range (C)	0.05–0.28	0.05–0.23	5×7 sponge pads
	EF Range (A)	0.05–0.33	0.07–0.23
	EF Peak	0.33	0.42
Adult 1 (Normal Anatomy)	EF Range (C)	0.11–0.30		5×5 sponge pads
	EF Range (A)	0.11–0.30
	EF Peak	0.36
Adult 2 (Normal Anatomy)	EF Range (C)	0.08–0.28		5×5 sponge pads
	EF Range (A)	0.07–0.24
	EF Peak	0.29
Adult 3 (Normal Anatomy)	EF Range (C)	0.04–0.19	0.09–0.18	5×5 sponge pads
	EF Range (A)	0.07–0.20	0.05–0.21
	EF Peak	0.23	0.21

*[A] denotes anode and [C] denotes cathode. Detailed descriptions of montages are contained in the text (Adapted from Kessler et al., 2013)*.

### Subject-specific dose decision

#### Intensity and duration

After all prior experience, constraints, and modeling considerations, it was determined that for this single-session intervention a current intensity of 0.7 mA for 10 consecutive minutes would be most appropriate to accomplish the primary purpose of the pilot—establishing tDCS safety and feasibility in children. The determination of the model for this child was based on the adult model. Current flow models in the adult present with a wide range of variability. For example, the potential exists for (1) paradoxical stimulation under the cathode (Batsikadze et al., 2013); (2) typical montages stimulating between the electrodes; and (3) accessing deeper structures (Dasilva et al., 2012). The low intensity decided upon was lower than an adult equivalent of approximately 1.0 mA. The intent was to prevent “direction flipping” of stimulation while at the same time to rely on available recent adult safety data with a concomitant assumption of similarities in electrophysiology in response to stimulation in the brains of both an adults and children (Brunoni et al., 2011; Marquez et al., 2013). Considering the child’s diagnosis of perinatal ischemic stroke, a challenge exists in attempts to incorporate into our calculations the lesion location and size as well as the variation in conductivity (cortex, cerebral spinal fluid) (Datta et al., 2009; Bikson et al., 2012). Incorporating neuromodulatory tools such as TMS as a locator for motor hotspots may provide additional knowledge regarding individual cortical excitability (Gillick et al., 2013).

#### Montage

Specific to the child with focal hemispheric lesion, a translational goal of the application of tDCS is to improve motor outcomes (Schlaug et al., 2008). Cathodal tDCS (M1-SO montage) has shown significant motor improvements in stroke, and specifically when coupled with rehabilitation (Nair et al., 2011). In children with language disorders, application of a similar montage-inferior frontal gyrus/contralateral SO montage was found to be safe and feasible. Although we found higher intensities within a range of comparable safety using an M1/SO montage (Figure 2), we decided upon a bihemispheric C3 contralesional cathodal/C4 ipsilesional anodal montage. Considering direct involvement of both hemispheres, we decided to investigate the safety and feasibility of tDCS intensity/application to the pediatric motor cortex bilaterally through an in-out lateralized pattern of activity between lesioned and non-lesioned hemispheres. This montage has been applied in both neurologically involved (e.g., stroke) and healthy adult populations to investigate enhancement of motor performance (Bolognini et al., 2011; Lefebvre et al., 2012; Kidgell et al., 2013b). We decided to maintain electrode sizes used in conventional (adult) trials (as opposed to “child size” electrodes), that combined with the use of low-voltage and reduced current intensity (0.7 mA) should enhance tolerability since current density at the electrode is low minimizing skin sensation and potential irritation. The stimulation was expected to be well tolerated given the total current (0.7 mA) selected to provide average adult brain electric fields (at 1.0 mA) and use of an electrode design validated for even higher intensities (up to 2.0 mA).

The goal of this study was to determine the intensity of stimulation and location of electrodes for this tDCS session in children with hemiparesis. After this integrative dose design consideration, including modeling, we determined that for this single-session intervention a current intensity of 0.7 mA in a bihemispheric C3 contralesional cathodal/C4 ipsilesional anodal montage for 10 consecutive minutes would be within historic safety limits based on generated brain electric fields to establish safety in tDCS application with translated to tolerable use in children. This bi-hemispheric montage over an M1-S0 montage was chosen to induce neuromodulation between the two hemispheres, lesioned and non-lesioned, with the potential to increase neuronal activity on the lesioned hemisphere and transiently inhibit over-activity of the nonlesioned hemisphere. The child tolerated this session well, with a sensation reported of mild, tolerable tingling under the electrodes within the first minute of stimulation which extinguished thereafter. The child reported no discomfort, nor did any adverse events occur (Gillick et al., in press).

## Discussion

The montage and parameters of dose represent a modification of historical adult methods facing the unknowns of pediatric stroke lesions and tDCS interventions. In summary, our aim was to design a tDCS protocol to determine an intervention applicable to pediatric stroke as there is evidence for efficacy of this application in adult stroke. We sought to determine this current intensity and montage allowing us to assess the electric field generation over the target region of M1. We used computational modeling and incorporation of past models to test the variation in parameters and decided upon those which supported safety and feasibility.

In progressing through a rational work-flow for montage selection, including assumptions about disease etiology and trade-offs in “best” montage design which require informed judgment based on integration across scientific and clinical tDCS literature we described our process. First, the study design started with safety as the primary objective. This requires a balance between minimizing dose (e.g., zero risk at zero dose) with maximizing dose to make results as relevant as possible for subsequent efficacy trials. This balance influences all subsequent decisions. Next, based on an assumption of asymmetric dysfunction we adopted a bi-cephalic (“lateralized”) approach. Then, though adult trials have used 2.0 mA, we recognized that clinical neurophysiologic studies increase non-monotonic dose response with high (2.0 mA) cathodal stimulation becoming excitatory. Based on our assumption of disease etiology and adopting a conservative approach, we elected to *approximate* a 1.0 mA adult dose. We also elected a relatively low duration. However both the intensity and duration determined were still within a range expected to produce significant lasting changes in brain excitability. Thereafter, to approximate electric field produced in adults, we determined it was necessary to decide which electric fields since the distribution in the brain is variable (e.g., electric field in target, average electric field, peak electric field across the brain). Moreover, in doing so we assumed no specific difference in susceptibility of the pediatric (injured) brain from adult in regards to safety or efficacy, but erred on approximating a “low” adult dose (intensity, duration). Finally, with the above caveats we elected to approximate a montage found successful in adult trials while recognizing the montage produced diffuse electrode flow through much of the brain, including deep brain structures. Reinforcing this approach, modeling predicted that the pattern of current flow across our subject was comparable to that in previous studies with adult subjects with no significant distortion due to the presence of the lesion.

Although this method may not be ideal, i.e., an individual representation of the *optimal* electric field generated at a target, the design of the model for this child attempted to incorporate the knowledge of tDCS modeling in adults and modify to a brain in a child with congenital hemiparesis. The models assume that the damaged brain regions have similar conductivity of cerebral spinal fluid while the peri-lesional area has the conductivity of healthy brain (Bijsterbosch et al., 2012). However, increased precision could incorporate changes in conductivity considering resultant gliosis or other pathologic processes that accompany cerebral lesions (Ruohonen and Karhu, 2012; Huang et al., 2014).

True comparability among future pediatric studies can only be established if each tDCS protocol articulates the rationale behind its methods, as well as current intensity, electrode size, location and stimulation duration (Nitsche et al., 2008; Batsikadze et al., 2013). Assessment of physiologic outcomes, serial applications and the longitudinal effects in combination with rehabilitation should include a thorough accounting of safety and dosing parameters.

## Conflict of interest statement

The authors declare that the research was conducted in the absence of any commercial or financial relationships that could be construed as a potential conflict of interest.
